# Nationwide Discrete Choice Experiment on Chinese Guardians’ Preferences for HPV Vaccination for Mothers and Daughters

**DOI:** 10.3390/vaccines12101186

**Published:** 2024-10-18

**Authors:** Jun Zhao, Tianshuo Zhao, Sihui Zhang, Ninghua Huang, Juan Du, Yaqiong Liu, Qingbin Lu, Chao Wang, Fuqiang Cui

**Affiliations:** 1 Department of Epidemiology and Biostatistics, School of Public Health, Peking University, Beijing 100191, China; zhao.jun@bjmu.edu.cn; 2 Department of Laboratorial Science and Technology & Vaccine Research Center, School of Public Health, Peking University, Beijing 100191, China; zts2018@pku.edu.cn (T.Z.); zhangsihui@bjmu.edu.cn (S.Z.); hnh@bjmu.edu.cn (N.H.); liuyaqiong@bjmu.edu.cn (Y.L.);; 3 Center for Infectious Diseases and Policy Research & Global Health and Infectious Diseases Group, Peking University, Beijing 100191, China; 4 Key Laboratory of Epidemiology of Major Diseases, Peking University, Ministry of Education, Beijing 100191, China; 5 Chinese Field Epidemiology Training Program, China CDC, Beijing 100191, China; 6 Beijing Jishuitan Hospital, Capital Medical University, Beijing 100191, China

**Keywords:** human papillomavirus vaccination, discrete choice experiment, Chinese guardians

## Abstract

**Background**: HPV vaccination is the key measure to prevent cervical cancer, but uptake in China lags behind global targets. Understanding Chinese guardians’ preferences is key to improving vaccine acceptance and coverage. **Methods**: A nationwide online discrete choice experiment survey was conducted among 4933 Chinese guardians across seven provinces in 2022 to quantify preferences and willingness to pay. Attributes included effectiveness, safety, duration, valency, location, and out-of-pocket cost. **Results**: Out of the 4933 guardians who participated in the study, 4179 (84.72%) were mothers. More than 60% of the guardians belonged to the age group of 35–44 years. Additionally, over half of the respondents (53.15%) had daughters between the ages of 9 and 14 years. Respondents were open to accepting the HPV vaccine with 95% efficacy and exceptional safety. Guardians expressed a preference for longer protection duration (specifically 15 years (βa = 0.340, 95% CI: 0.31, 0.37)) and higher vaccine valency. As for willingness to pay, the respondents placed the highest value on vaccine efficacy, being willing to shell out more than USD 1100 for 95% protection as compared to 50%. Furthermore, very good safety commanded a premium of over USD 800 when compared to average safety. When it comes to willingness to uptake, a vaccine with 95% efficacy led to a more than 35% increase in acceptance as compared to one with 50% efficacy. Similarly, exceptional safety resulted in an increased willingness to uptake of over 25% when compared to average safety. **Conclusions**: The DCE highlighted effectiveness, safety, and durability as critical drivers of HPV vaccine acceptance, but substantial barriers persist regarding adolescent female coverage in China.

## 1. Introduction

Infection with human papillomavirus (HPV) has been conclusively linked to the etiology of cervical cancer, and burgeoning research indicates its significance in the pathogenesis of other anogenital malignancies, such as cancers of the anus, vulva, vagina, and penis, as well as oropharyngeal neoplasms [1,2,3,4,5]. From an epidemiological standpoint, the global prevalence of HPV is substantial, with cervical cancer ranking as the second most prevalent malignancy in women aged 15 to 44 years [4]. Current estimates indicate that every year, 604,127 women are diagnosed with cervical cancer, and 341,831 died from the disease in 2024. Approximately 18.2% of the global burden of cervical cancer in 2020 occurred in China [6,7].

Prophylactic vaccination against HPV, along with screening and treatment of pre-cancer lesions, have proven to be effective preventive measures against HPV-related diseases and are highly cost-effective. The World Health Organization promotes the integration of the HPV vaccine into national immunization programs, highlighting the optimal efficacy of preventing cervical cancer through vaccinating girls prior to their initiation of sexual activity [8]. However, the implementation of the vaccine in low-income and middle-income countries, where the majority (90%) of cervical cancer deaths occur, lags behind that in high-income countries. To address this disparity, the WHO has established targets, commonly referred to as the 90–70–90 targets, aiming to eliminate cervical cancer as a public health problem by the year 2030, which includes vaccinating 90% of girls by the age of 15 [8]. Nevertheless, the attainment of widespread HPV vaccination coverage has encountered various obstacles, such as exorbitant expenses and vaccine hesitancy, which have contributed to insufficient global uptake [9]. Guardians make the majority of healthcare decisions for girls, and mothers have a significant influence on the vaccinations of their daughters [10]. A study by Goldstein and Weber [11] indicated that individuals apply different strategies when deciding for themselves or someone else. Perceived sexual inactivity of adolescents, insufficient knowledge about vaccination timing, safety and effectiveness and preference for the 9vHPV vaccine influenced parents’ individual decisions and were consistent with determinants of vaccine hesitancy [12]. HPV vaccine hesitancy is common among guardians of secondary school girls in mainland China; nearly one-third of the guardians had never heard of or considered vaccinating their children against HPV, and vaccine shortage and busy schedules were the main barriers preventing implementation among the guardians who had already decided to vaccinate their children [13].

China is a latecomer in marketing the first HPV vaccine, but it takes its place in the front ranks of the world for R&D on HPV vaccines [14]. To date, five HPV vaccines have been approved in China: Cervarix, Gardasil, Gardasil 9, Cecolin, and Walrinvax [15]. However, the national immunization program (NIP) in China has not included the HPV vaccine, and supply remains limited and costly, so getting an HPV vaccine is completely self-pay for age-eligible females. This creates many access barriers, such as the tight supply of the HPV vaccine (especially the 9-valent vaccine) and the economic burden for many Chinese families for their age-eligible girls to receive a complete dose of HPV vaccination (especially the 9-valent vaccine). In addition, HPV vaccines are only approved for females, with RCTs in males ongoing. Therefore, as of 2024, there was no approved HPV vaccination program for males. As a result of these limitations, population coverage is currently low, with only 3% of females vaccinated three years after HPV vaccine licensure in China [16].

This subpar vaccination rate falls well below the WHO’s objective of achieving a 90% coverage of HPV vaccination among all adolescent girls by 2030. A recent report in 2021 pointed out that the coverage rate of the HPV vaccine for adolescents was lower than 3%, while the coverage for the entire population was below 6% [17]. Several factors contribute to this situation, including limited vaccine availability, high costs associated with the vaccine, and a general lack of public awareness regarding HPV and its corresponding vaccines [16]. For individuals, they also consider a set of alternatives based on their needs and interests to arrive at a vaccine decision [18].

In order to effectively overcome the obstacles hindering the acceptance of vaccines and promote the utilization of the HPV vaccine, it is crucial to comprehensively understand the preferences of the intended vaccine recipients in relation to the various attributes of HPV vaccines. In China, the primary decision makers for girls’ healthcare are their guardians, and a study conducted in New Zealand also demonstrated that mothers exert a significant influence on their daughters’ vaccination decisions [10]. To uncover and quantify the preferences of both daughters and guardians toward a potential HPV vaccine, we employed the discrete choice experiment (DCE) methodology. Pharmaceutical companies and health policymakers can enhance acceptance and expand vaccine coverage of HPV vaccination initiatives by showcasing the vaccine attributes that are highly valued by the target demographic. This study represents a significant stride towards the realization of the WHO’s 90–70–90 targets by 2030.

## 2. Materials and Methods

### 2.1. Study Design

We conducted a cross-sectional, population-based online survey among the Chinese population using a structured questionnaire from 6 June to 17 September 2022. This research was registered with the European Union electronic register of Post-Authorisation Studies (EU PAS Register^®^, identifier: EUPAS1000000200). It was an online survey for the population aligned with the inclusion criteria for those residing in China. The online survey was managed by the study group conducted through local partners and distributed in 12 locations among seven provinces. Provinces were selected primarily based on the balance of geographical and economic distribution, and the chosen provinces were Shandong province and Zhejiang province in east China; Anhui, Henan, and Heilongjiang provinces in the middle; and Sichuan and Gansu provinces in the west. Participants could complete the survey questionnaire, which took approximately three minutes on average, either by mobile phone or computer. Data were collected on Wen Juan Xing (Changsha Ranxing Information Technology Co., Ltd., Changsha, China), a widely used online platform embedded in WeChat providing online questionnaire design and survey functions equivalent to Amazon Mechanical Turk, Qualtrics, SurveyMonkey, or CloudResearch. Importantly, its personal identification function allows for an authentic, diverse, and representative sample. Informed consent was sent through the same survey link. All participants had to fill out the informed consent to complete the survey.

### 2.2. Sample Recruitment and Data Collection

Twelve sites from seven representative provinces were selected for the questionnaire survey. The inclusion criteria for guardians were (a.) those with daughter between the ages of 9 and 18 years, (b.) who were local resident for at least 6 months, (c.) whose daughters had no history of major illness or hormone therapy, and (d.) who completed the informed consent and complied with all research procedures. The structured questionnaire contained information on demographic characteristics, including parents’ relationship with the daughter, age, education, residence, family, and occupation, as well as their daughters’ age and grades, and discrete choice experiment (DCE) scales (questionnaire in Supplementary Materials).

### 2.3. Discrete Choice Experiment Scales

Based on literature reviews, interviews with experts with several years of vaccination experience, as well as focus group discussions, six attributes (protective effect, safety, effect duration, production location, vaccine valency, and out-of-pocket cost of vaccination and their levels) were finally included within the DCE [15,19,20,21]. The consideration of the above six attributes was primarily based on the WHO-released Vaccine Hesitancy 3C model [22]. Protective effect and safety belong to the confidence dimension, while effect duration, vaccine type, and full payment belong to the convenience dimension. The subjectivity of the complacency dimension has not been classified as an objective attribute for consideration. Location (import or local production) was recommended to be added based on expert advice.

Secondly, a DB-efficient design was developed using the Ngene software (version: 1.1.2, Sydney, Australia), which yielded 24 choice sets that were further divided into 12 blocks to reduce respondents’ cognitive burden. Accordingly, 24-fold observations were gathered for each respondent. To check for internal consistency, one choice set in each block was duplicated and was excluded from the analysis.

### 2.4. Quality Control

To control for randomness, we employed a cluster sampling method by selecting schools from 12 cities across seven representative provinces in China encompassing both inland and coastal areas with varying industrial structures. This approach ensured randomness through a large sample size and cluster randomization based on class.

Regarding confidentiality and anonymity, we implemented stringent measures to safeguard participants’ information. The data collection was conducted in an anonymous manner, ensuring that no personally identifiable information was linked to the survey responses. Participants were informed that their identities would remain completely confidential, and aliases were used to further protect their identities throughout the research process. This assurance fostered an environment where respondents felt secure to provide honest answers. Furthermore, all survey data were securely stored and only accessible to the research team, in alignment with ethical research guidelines.

We monitored the progress of the survey every day. After the deadline, we checked the accuracy of the data. A quality control question (What year is this year?) was set for detecting inattentive samples, and all records passed this question. Answers from duplicate IP addresses were determined based on the first record, and the following records were excluded (n = 6). Moreover, questionnaires were excluded if (1) the IP addresses were outside the mainland of China (n = 1) or (2) there were logical contradictions between the answers to the questionnaire (n = 0).

### 2.5. Statistical Analysis

The original questionnaire involved the unit of measurement for the payment amount in CNY. This article has converted this to USD based on the exchange rate of 7.1 on 5 August 2024. Categorical variables are expressed as absolute and relative frequencies for different groups. The continuous variables were re-classified into categorical variables, with three categories according to the distribution. For example, age was re-classified into 25–34 years, 35–44 years, and 45–55 years. The conditional logit model was employed to analyze the guardians’ willingness for mothers’ HPV vaccination or their daughters’ vaccination. In addition, the model fitness was compared from multiple perspectives. Willingness to pay based on the attributes of HPV vaccines was calculated based on Formula (1), and willingness to uptake HPV vaccines of different attributes was calculated based on Formula (2). SAS (version 9.4, Cary, NC, USA) was used for data cleaning and statistical analysis. The significance level was set at a *p* value of less than 0.05.

(1) $${WTP}_{x}=-\frac{\beta_{X}}{\beta_{cost}}\times 1000$$

(2) $$P_{i}=\frac{e^{\beta \times x_{i}}}{e^{\beta \times x_{i}}+e^{\beta \times x_{j}}}$$

## 3. Results

### 3.1. The Demographic Characteristics of Study Participants

The study comprised 4933 guardians, with the predominant majority (84.72%) being mothers. A significant proportion, exceeding 60%, belonged to the age group of 35–44 years. Furthermore, more than half (53.15%) of these guardians had daughters between the ages of 9 and 14 years. Among the guardians, the majority (55.77%) were employed in enterprises, and a significant portion (58.99%) had attained an educational level of junior high school or below. The daughters of the guardians were evenly distributed across grades 4 to 12. Approximately 40% of the guardians reported a monthly family income below USD 423, with a substantial 70.30% residing in rural areas (Table 1 for details).

### 3.2. Willingness for Guardians’ HPV Vaccination

The logit model analyzed the willingness of 4933 guardians to obtain the HPV vaccine for themselves or their wives, with a total of 118,392 observations considered. The results indicated that vaccine effectiveness was a significant driver of willingness, with an adjusted coefficient (βa) of 0.36 (95% CI: 0.33, 0.39; *p* < 0.001) for a 75% effective vaccine and βa of 0.73 (95% CI: 0.71, 0.76; *p* < 0.001) for a 95% effective vaccine when compared to a 50% effective vaccine. Furthermore, good and very good vaccine safety had βa values of 0.49 (95% CI: 0.45, 0.53; *p* < 0.001) and 0.54 (95% CI: 0.50, 0.57; *p* < 0.001), respectively, when compared to average safety. A longer duration of vaccine effect, specifically 15 years (βa = 0.340, 95% CI: 0.31, 0.37) and lifelong (βa = 0.55, 95% CI: 0.53, 0.58), was preferred over a 5-year duration (*p* < 0.001). Additionally, broader vaccines, including a 4-valent vaccine (βa = 0.23, 95% CI: 0.20, 0.26) and a 9-valent vaccine (βa = 0.28, 95% CI: 0.25, 0.31), were preferred over a 2-valent vaccine (*p* < 0.001). Subgroup analysis showed guardians living in rural and urban areas had similar preferences for the attributes studied, except for exhibiting opposite directions towards imported vaccines (βa: −0.07 vs. 0.05) (Table 2 for details).

### 3.3. Willingness for Daughters’ HPV Vaccination

The logit model examined 4933 guardians’ willingness for HPV vaccination of their daughters across 118,392 observations. Vaccine effectiveness significantly increased willingness, as the 75% effective vaccine had a βa of 0.36 (95% CI: 0.33, 0.39; *p* < 0.001), and the 95% effective vaccine had a βa of 0.75 (95% CI: 0.72, 0.77; *p* < 0.001) compared to a 50% effective vaccine. Good and very good vaccine safety had βa values of 0.50 (95% CI: 0.46, 0.54; *p* < 0.001) and 0.55 (95% CI: 0.52, 0.59; *p* < 0.001) versus average safety. Longer duration of vaccine effects (15 years βa = 0.34, 95% CI: 0.33, 0.38; lifelong βa = 0.52, 95% CI: 0.49, 0.55) were preferred over 5 years (*p* < 0.001). Broader vaccines (4-valent βa = 0.20, 95% CI: 0.17, 0.24; 9-valent βa = 0.34, 95% CI: 0.31, 0.37) were preferred versus the 2-valent vaccine (*p* < 0.001). The subgroup analysis showed guardians living in rural and urban areas had similar preferences for the attributes studied, except for exhibiting opposite directions towards imported vaccines (βa: −0.07 vs. 0.05) (Table 3 for details).

### 3.4. Willingness to Pay Based on the Attributes of HPV Vaccines

Guardians exhibited a willingness to pay USD 565 for 75% vaccine effectiveness and USD 1147 for 95% effectiveness when vaccinating mothers. However, when considering the vaccination of their daughters, the willingness to pay was nearly threefold higher at USD 1710 for 75% effectiveness and USD 3498 for 95% effectiveness. Furthermore, guardians indicated a willingness to pay USD 767 for good safety and USD 842 for very good safety when vaccinating mothers, while the figures rose to USD 2347 for good safety and USD 2596 for very good safety when vaccinating daughters.

In terms of the duration of vaccine effect, guardians were prepared to pay USD 532 for 15 years and USD 867 for lifelong protection when vaccinating themselves (or their wives), with the willingness to pay increasing to USD 1676 for 15 years and USD 2441 for lifelong duration when considering their daughters. With regard to the breadth of protection, guardians were willing to pay an additional USD 362 for a 4-valent vaccine and USD 430 for a 9-valent vaccine, compared to a 2-valent vaccine, for themselves (or their wives). Conversely, their willingness to pay for their daughters was notably higher, with an additional USD 953 for a 4-valent vaccine and USD 1615 for a 9-valent vaccine.

To provide more nuanced results, we also conducted subgroup analyses based on guardians’ education levels and family income. These subgroup analyses revealed that as education levels and family income increased, there was a corresponding rise in the willingness to pay for the HPV vaccine. However, it was consistently observed that guardians’ willingness to pay for their daughters’ vaccination remained significantly higher than their willingness to pay for their own vaccination (Table 4 for details).

### 3.5. Willingness to Uptake HPV Vaccines of Different Attributes

**Figure 1:**
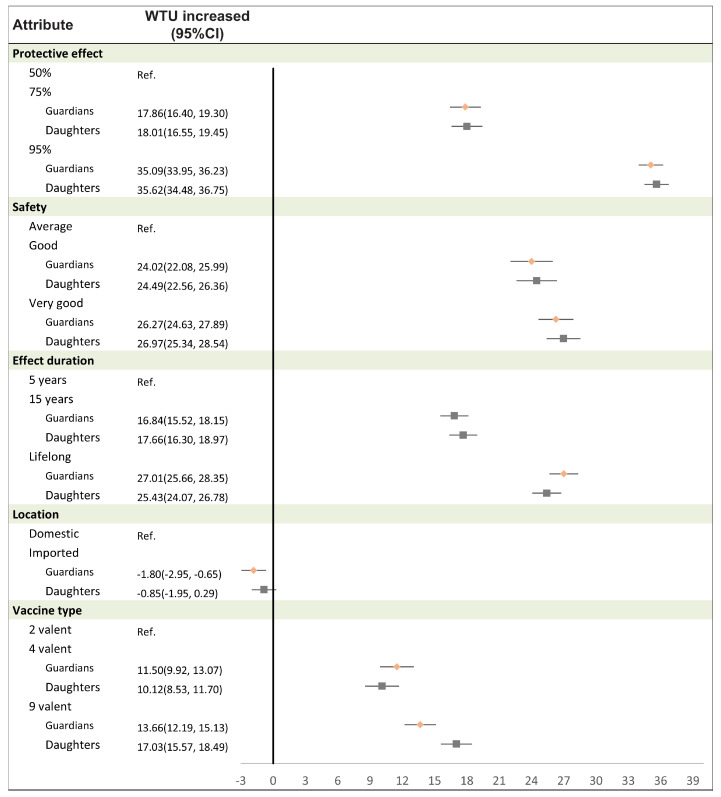
Willingness to uptake (WTU) based on the attributes of HPV vaccines.

Comparing the willingness to uptake (WTU) between mothers and daughters, the impacts were remarkably similar. For instance, when comparing a vaccine with 95% versus 50% efficacy, the WTU increased by 35.09% (95% CI: 33.95–36.23) for mothers and 35.62% (95% CI: 34.48–36.75) for daughters. Similarly, a vaccine perceived as very good versus average safety increased the WTU by 26.27% (95% CI: 24.63–27.89) for mothers and 26.97% (95% CI: 25.34–28.54) for daughters. Furthermore, a lifelong versus 5-year duration of protection led to a 27.01% (95% CI: 25.66–28.35) WTU increase for mothers and 25.43% (95% CI: 24.07-26.78) for daughters. The only notable difference was observed for a 9-valent versus 2-valent vaccine, which increased the WTU by 13.66% (95% CI: 12.19–15.13) for mothers, as opposed to 17.03% (95% CI: 15.57–18.49) for daughters. Notably, vaccine production location showed little influence on the willingness to uptake for either mothers or daughters (Figure 1 for details).

## 4. Discussion

This study illuminated Chinese guardians’ preferences and willingness to pay for HPV vaccine attributes to prevent cervical cancer in themselves/their wives and daughters. Effectiveness, safety, duration, valency, and affordability significantly influenced willingness to vaccinate. It is worth pointing out that guardians were willing to pay over 50% more across all metrics to protect their daughters compared to themselves, underscoring an urgent need to prioritize female adolescent vaccination.

Guardians demonstrated clear rational prioritization of vaccine effectiveness as the most valued attribute, consistent with multiple former experiments globally [23,24,25]. As the fundamental premise of any vaccine is providing protection against its target disease, higher effectiveness logically engenders greater willingness to accept and pay. Guardians were willing to pay over USD 1100 more for maximal 95% versus 50% protection. This indicates that suboptimal effectiveness could significantly deter uptake. Our findings aligned with a recent study showing that Chinese adults ranked effectiveness among the most important drivers of COVID-19 vaccination choices [26]. Lisa A. Prosser [27] directly compared vaccination preferences for adult and child vaccination against COVID-19 using a discrete choice experiment approach and found that vaccine effectiveness was a significant attribute, followed by the risk of rare severe adverse events. Results were similar when comparing choices across adult and child vaccination, with a slightly stronger preference for fewer severe adverse events and full regulatory approval in children compared with adults.

Reassurance of a high safety profile was another prerequisite, echoing public vaccine sentiments amid recent scandals in China. Guardians had over USD 800 higher willingness to pay for “very good” versus average safety vaccines. It is probable that apprehensions regarding the well-being of their daughters were amplified, as guardians were prepared to pay over USD 2500 more for maximum safety assurances for their daughters compared to protection for themselves. This highlights that safety doubts could substantially undermine adolescent female vaccination initiatives without proper transparency and risk communication.

Lifelong protection was also clearly favored, conferring an additional USD 850 premium versus short-term 5-year coverage for guardians themselves and over USD 2400 more for guardians’ daughters. Parents inherently seek to safeguard children’s welfare far into the future. Furthermore, with evidence that even one HPV vaccine dose strongly shields against cervical cancer for at least 10 years, incomplete regimens still offer meaningful protection [28,29,30]. Hence, governments must strategize long-term vaccination financing.

Additionally, guardians were willing to pay over USD 360 more for multivalent vaccines offering prevention against more high-risk HPV strains for themselves, and over USD 950 more for daughters. This aligns with the greater risk-reduction afforded by higher-valency vaccines. Cost unsurprisingly posed barriers, though guardians exhibited willingness to pay given adequate vaccine attributes. Collectively, our findings showcase that guardians conduct rational risk–benefit calculations centered on effectiveness, safety, and durability.

Remarkably, guardians consistently exhibited over twice the willingness to pay for maximal protections for daughters versus self-protection. This clashes against patterns of mothers downplaying daughters’ susceptibility to “adult” threats like HPV. However, in sexually conservative cultures like China, there has been a growing tolerance towards premarital sexual behavior, and adolescents are increasingly engaging in sexual activities at an earlier age [31,32]. HPV remains widely perceived as a taboo STI [33], and considering that the HPV vaccine is best given before the onset of sexual activity [34], this could potentially heighten parents’ anxieties of infection risk and desire to shield teenage daughters. Additionally, the heavy cervical cancer burden in China and distressed finances from exorbitant cancer care costs could compound worries of daughter’s futures [35]. These insights spotlight that young girls must be the priority focus for vaccination strategies in China to overturn norms and ensure coverage.

Our findings have meaningful implications. The willingness-to-pay quantification offers guidance for policymakers to optimize vaccine pricing and subsidies at the intersection of affordability and profitability to enable uptake. Additionally, public education should broadcast information on vaccine effectiveness, safety data, and duration of immunity using transparent communication to address parents’ questions and concerns. Finally, political will is essential to enact financing policies and logistical plans centered around adolescent females. Considering the similar preferences in both rural and urban areas and the accessibility of HPV vaccines in rural areas [36], it is necessary to improve vaccine distribution channels or address healthcare access issues in rural areas. Providing vaccination subsidies, improvements in vaccine supply chains, healthcare infrastructure, or long-term funding mechanisms can help improve the accessibility and vaccination rate of HPV vaccines for Chinese women. Overall, this study delivers actionable evidence for China to attain the WHO’s cervical cancer elimination goals.

While this study provides meaningful insights into Chinese guardians’ preferences for HPV vaccination, some limitations should be considered when interpreting the results. Firstly, the use of an online survey means that the authenticity of participants’ personal information cannot be verified, and selection bias and survey bias could not be ignored, since those who do not have access to the internet and who lack social desirability in self-reported willingness may have had different preferences for HPV vaccination. Additionally, the non-probability sampling method could introduce clustering around groups with a higher education, younger ages, and urban regions. We sought to mitigate this by recruiting participants from diverse areas, but it still limits generalizability. Regarding the discrete choice experiment methodology itself, this study employed a non-full factorial design, meaning not all possible vaccine profiles were compared. Using labeled hypothetical vaccines also reduces realism, which may introduce a potential deviation from real market settings, which is often referred as hypothetical bias [37]. Future studies should examine preferences using real marketed HPV vaccines to increase applicability. Additionally, vaccine attributes were constrained; logistical considerations like dosing schedules could plausibly influence choices. Finally, the descriptions used for side effect levels (low/medium/high) were subjective without precise definitions. This could allow personal interpretations to affect choices.

Overall, while this nationwide study collected a large sample revealing meaningful trends in HPV vaccine preferences, the limitations warrant consideration. Further research with randomized recruitment, full factorial designs comparing real vaccines, and tightly defined attributes could confirm and extend the results. A cost–benefit analysis and trials thus remain vital to evaluate the impact of acting upon the preferences found here to shape real policies and programs. This could provide direct evidence guiding translation of these insights to improve HPV vaccination adoption at scale in China.

## 5. Conclusions

In summary, this comprehensive DCE provided a robust method for elucidating the priorities and willingness to pay of Chinese guardians for HPV vaccination. The results notably emphasize the significance of vaccine effectiveness and safety, longer duration, increased valency, and reduced cost barriers as crucial determinants for vaccination. Among these, the priority should be given to emphasizing and educating about vaccine effectiveness and safety. Importantly, guardians placed a significantly higher value on protecting their daughters over themselves, highlighting the urgent need to develop policies that ensure high HPV vaccine coverage in adolescent girls. We also reported subgroup analyses that show how the results vary by residence, family income, and education level. These new and more nuanced insights form a valuable foundation for reshaping communication strategies, pricing policies, and political initiatives to enhance preferences, promote vaccination, and reduce infections, considering the current satisfactory effectiveness and safety of HPV vaccines.

## Figures and Tables

**Table 1:** The demographic characteristics of study participants.

Characteristics		N	%
Relationship with daughter	Mother	4179	84.72
	Father	754	15.28
Age	25–34	527	10.68
	35–44	3138	63.61
	45–55	1268	25.70
Daughter’s age	9–14	2622	53.15
	15–18	2311	46.85
Occupation	Enterprise staff	2751	55.77
	Farmers	2182	44.23
Education	Junior high school or below	2910	58.99
	Senior high school	734	14.88
	Bachelor or above	1289	26.13
Daughter’s grade	Grade 4	551	11.17
	Grade 5	446	9.04
	Grade 6	543	11.01
	Grade 7	370	7.50
	Grade 8	480	9.73
	Grade 9	624	12.65
	Grade 10	659	13.36
	Grade 11	605	12.26
	Grade 12	655	13.28
Family income	<423	2003	40.60
	423–704	1811	36.71
	>704	1119	22.68
Residence	Rural	3468	70.30
	Urban	1465	29.70

**Table 2:** Conditional logit model of the guardians’ willingness for guardians’ HPV vaccination.

Attribute	Levels	β	95% CI_c_	95% CI_a_	*p*	Rural		Urban	
						β	95% CI_c_	β	95% CI_c_
Protective effect	50%	Ref.				Ref.		Ref.	
	75%	0.36	(0.33, 0.39)	(0.33, 0.39)	<0.001	0.35	(0.32, 0.39)	0.38	(0.33, 0.44)
	95%	0.73	(0.71, 0.76)	(0.71, 0.76)	<0.0001	0.72	(0.69, 0.75)	0.77	(0.72, 0.81)
Safety	Average	Ref.				Ref.		Ref.	
	Good	0.49	(0.45, 0.53)	(0.45, 0.53)	<0.001	0.50	(0.45, 0.55)	0.47	(0.39, 0.54)
	Very good	0.54	(0.50, 0.57)	(0.50, 0.57)	<0.001	0.55	(0.51, 0.59)	0.51	(0.45, 0.57)
Effect duration	5 years	Ref.				Ref.		Ref.	
	15 years	0.34	(0.31, 0.37)	(0.31, 0.37)	<0.001	0.34	(0.30, 0.37)	0.35	(0.30, 0.40)
	Lifelong	0.55	(0.53, 0.58)	(0.53, 0.58)	<0.001	0.59	(0.55, 0.62)	0.48	(0.43, 0.53)
Location	Domestic	Ref.				Ref.		Ref.	
	Imported	−0.04	(−0.06, −0.01)	(−0.06, −0.01)	0.002	−0.07	(−0.10, −0.05)	0.05	(0.01, 0.09)
Vaccine type	2-valent	Ref.				Ref.		Ref.	
	4-valent	0.23	(0.20, 0.26)	(0.20, 0.26)	<0.001	0.24	(0.20, 0.28)	0.21	(0.15, 0.27)
	9-valent	0.28	(0.25, 0.31)	(0.25, 0.31)	<0.001	0.27	(0.24, 0.31)	0.28	(0.22, 0.33)
Full payment		−0.09	(−0.10, −0.08)	(−0.10, −0.08)	<0.001	−0.10	(−0.11, −0.09)	−0.06	(−0.08, −0.05)

Notes: CI_c_, the crude coefficient of confidence interval; CI_a_, the adjusted coefficient of confidence interval by age, gender, income, education, daughter’s grade, and residence; Respondents = 4933; Number of observations = 118,392; Prob > chi-square < 0.0001; Log-likelihood = 82,063.081; Pseudo R^2^ = 0.04.

**Table 3:** Conditional logit model of the guardians’ willingness for their daughters’ HPV vaccination.

Attribute	Levels	β	95% CI_c_	95% CI_a_	*p*	Rural		Urban	
						β	95% CI_c_	β	95% CI_c_
Protective effect	50%	Ref.				Ref.		Ref.	
	75%	0.36	(0.33, 0.39)	(0.33, 0.39)	<0.001	0.35	(0.32, 0.39)	0.39	(0.34, 0.45)
	95%	0.75	(0.72, 0.77)	(0.72, 0.77)	<0.001	0.72	(0.69, 0.75)	0.80	(0.75, 0.85)
Safety	Average	Ref.				Ref.		Ref.	
	Good	0.50	(0.46, 0.54)	(0.46, 0.54)	<0.001	0.51	(0.46, 0.56)	0.49	(0.41, 0.56)
	Very good	0.55	(0.52, 0.59)	(0.52, 0.59)	<0.001	0.57	(0.52, 0.61)	0.52	(0.46, 0.59)
Effect duration	5 years	Ref.				Ref.		Ref.	
	15 years	0.34	(0.33, 0.38)	(0.33, 0.38)	<0.001	0.35	(0.31, 0.38)	0.38	(0.33, 0.44)
	Lifelong	0.52	(0.49, 0.55)	(0.49, 0.55)	<0.001	0.55	(0.52, 0.59)	0.45	(0.40, 0.50)
Location	Domestic	Ref.				Ref.		Ref.	
	Imported	−0.02	(−0.04, 0.01)	(−0.04, 0.01)	0.151	−0.05	(−0.08, −0.03)	0.07	(0.03, 0.11)
Vaccine type	2-valent	Ref.				Ref.		Ref.	
	4-valent	0.20	(0.17, 0.24)	(0.17, 0.24)	<0.001	0.20	(0.16, 0.24)	0.20	(0.14, 0.26)
	9-valent	0.34	(0.31, 0.37)	(0.31, 0.37)	<0.001	0.32	(0.29, 0.36)	0.40	(0.34, 0.45)
Full payment		−0.03	(−0.04, −0.02)	(−0.04, −0.02)	<0.001	−0.04	(−0.05, −0.03)	0.00	(−0.02, 0.02)

Notes: Notes: CI_c_, the crude coefficient of confidence interval; CI_a_, the adjusted coefficient of confidence interval by age, gender, income, education, daughter’s grade, and residence; N = 4933; Number of observations = 118,392; Prob > chi-square < 0.0001; Log-likelihood = 82,063.081; Pseudo R^2^ = 0.0481.

**Table 4:** Willingness to pay (WTP) and 95% CI based on the attributes of HPV vaccines.

Attribute	All	Education			Family Income		
		Junior High School or Below	Senior High School	Bachelor or Above	<423	423–704	>704
Protective effect							
50%							
75%							
Guardians	565 (518, 612)	462 (409, 515)	755 (603, 905)	806 (683, 928)	369 (316, 419)	583 (501, 663)	2165 (1843, 2483)
Daughters	1710 (1568, 1850)	1131 (1002, 1258)	4133 (3319, 4937)	7820 (6634, 8996)	854 (741, 969)	1928 (1656, 2197)	3313 (2810, 3816)
95%							
Guardians	1147 (1106, 1188)	946 (901, 991)	1460 (1332, 1589)	1650 (1544, 1756)	759 (714, 804)	1210 (1139, 1282)	4172 (3893, 4453)
Daughters	3498 (3376, 3620)	2323 (2213, 2430)	7934 (7233, 8644)	16,456 (15,430, 17,477)	1701 (1605, 1795)	4182 (3946, 4422)	6520 (6081, 6962)
Safety							
Average	Ref.						
Good							
Guardians	767 (703, 833)	668 (597, 740)	889 (685, 1093)	1034 (867, 1201)	486 (415, 557)	813 (702, 923)	2939 (2498, 3378)
Daughters	2347 (2155, 2535)	1607 (1433, 1778)	4948 (3843, 6042)	10,753 (9134, 12,362)	1103 (946, 1260)	2766 (2398, 3133)	4653 (3961, 5344)
Very good							
Guardians	842 (787, 897)	743 (683, 804)	951 (777, 1124)	1118 (975, 1260)	540 (480, 600)	901 (806, 995)	3127 (2747, 3507)
Daughters	2596 (2432, 2756)	1844 (1696, 1990)	5167 (4211, 6119)	11,244 (9872, 12,628)	1260 (1127, 1394)	3063 (2750, 3377)	4889 (4304, 5467)
Effect duration							
5 years	Ref.						
15 years							
Guardians	532 (490, 574)	433 (386, 481)	705 (569, 840)	765 (656, 876)	333 (286, 380)	553 (478, 627)	2144 (1843, 2443)
Daughters	1676 (1545, 1803)	1065 (950, 1180)	4078 (3307, 4843)	8233 (7134, 9339)	1292 (1183, 1402)	2938 (2677, 3196)	3827 (3339, 4314)
Lifelong	Ref.						
Guardians	867 (822, 912)	792 (742, 842)	998 (855, 1142)	1038 (920, 1157)	635 (586, 685)	903 (824, 980)	2660 (2348, 2968)
Daughters	2441 (2305, 2577)	792 (742, 842)	998 (855, 1142)	1038 (920, 1157)	1292 (1183, 1402)	2938 (2677, 3196)	3827 (3339, 4314)
Location							
Domestic	Ref.						
Imported							
Guardians	−56 (−92, −20)	−111 (−150, −71)	−9 (−123, 104)	109 (14, 202)	−90 (−130, −52)	−22 (−83, 40)	63 (−179, 301)
Daughters	−80 (−184, 28)	−190 (−285, −93)	248 (−379, 866)	1105 (199, 2011)	−99 (−185, −14)	−107 (−310, 101)	259 (−121, 634)
Vaccine type							
2 valent	Ref.						
4 valent							
Guardians	362 (311, 412)	351 (295, 406)	342 (180, 505)	411 (282, 543)	263 (207, 319)	377 (289, 464)	1154 (803, 1503)
Daughters	953 (803, 1103)	714 (579, 847)	1550 (674, 2417)	4004 (2738, 5258)	494 (372, 619)	1160 (867, 1451)	1554 (1006, 2107)
9 valent	Ref.						
Guardians	430 (383, 477)	380 (327, 433)	519 (372, 667)	546 (423, 668)	267 (216, 319)	474 (393, 556)	1613 (1292, 1932)
Daughters	1615 (1474, 1756)	1041 (914, 1170)	3548 (2737, 4355)	8156 (6975, 9338)	753 (639, 867)	1963 (1695, 2236)	3126 (2625, 3632)

## Data Availability

Original data are available on request. These were stored on password-protected computers at the Department of Laboratorial Science and Technology & Vaccine Research Center, School of Public Health, Peking University. Readers who wish to gain access to the data can write to the corresponding author.

## Supplementary Materials

The following supporting information can be downloaded at: https://www.mdpi.com/article/10.3390/vaccines12101186/s1, Questionnaire: Study on the difference between HPV vaccination rates and their influencing factors among school-aged females ages 9–18 years old in China.
