# Supplementary material for: Nationwide Discrete Choice Experiment on Chinese Guardians’ Preferences for HPV Vaccination for Mothers and Daughters
Source: Vaccines (Basel). 2024 Oct 18;12(10):1186. doi: 10.3390/vaccines12101186 (PMC11512336; doi:10.3390/vaccines12101186)
Supplement: Supplementary file 1 [file vaccines-12-01186-s001.zip › vaccines-3180215-supplementary.pdf]

Study on the difference between HPV vaccination rates and its influence factors among school females aged 9-18 years old in China

Hello! We are researchers at the School of Public Health, Peking University. We are conducting a study on difference between HPV vaccine rates and its influence factors among school females aged 9-18 years old in China, and you meet the enrollment criteria of this study, so we would like to invite you to take part in this study.

In this study, the questionnaire will be used to collect your information about your daughter's HPV vaccination, disease history, genetic history, etc., and to quantitatively measure the knowledge, attitude, confidence, and willingness to be vaccinated against HPV vaccine.

Some of the questions in the questionnaire may make you feel uncomfortable and you may refuse to answer. In order to control these risks, the study will keep what you fill in confidential and will not have an adverse effect on the work, study, or life of the survey participants. There is no risk that the study will expose you to civil or criminal liability and there will be no harm to your financial situation, employability or reputation.

Your participation in this study is entirely voluntary. If you do not want to, you can refuse to participate, and this will not have any negative effect on your current or future medical care. Even after you agree to participate, you may change your mind at any time and tell the researcher to withdraw from the study, and your withdrawal will not affect your ability to get regular medical care.

The full text of the informed consent form is available at
https://docs.qq.com/doc/DWFRhanNLZWxzVHN3

The project leader for this study is Fuqiang Cui (School of Public Health, Peking University).
If you have any questions related to this study, please contact:
Fuqiang Cui, School of Public Health, Peking University/13718891696
Chao Wang, School of Public Health, Peking University/13522189226

Have you read the informed consent form and agree to participate in this study? [Single choice question] *

○ Yes

○ No

Instructions for filling in the questionnaire:

1. Please fill in the real situation;
2. Please refer to the ID card for the date of birth;
3. Please make sure that the code in the questionnaire is the same for me and my daughter, which are the last 5 digits of the same cell phone number;

I. Basic information

2. Please select provincial cities and districts: [fill in the blanks]*

____

3. Your relationship with your daughter is [single choice]*

○Mother and Daughter

○Father and daughter

4. The year of your birth is ______ and the year of your daughter's birth is ______ [fill in the blanks] *

5. Your ethnicity is [single choice]*

○The Han nationality

○Other ________________ *

6. Your occupation is [single choice]*

○Enterprise staffs

○Farmers

7. Your educational qualifications are [single choice]*

Junior high school or below

Senior high school

Bachelor or above

8. Your daughter's grade level is [Single choice]*

Grade 4

Grade 5

Grade 6

Grade 7

Grade 8

Grade 9

Grade 10

Grade 11

Grade 12

9. Monthly family income (total monthly household income ÷ total number of persons in the household) [Single choice]*

○3000 RMB or less

○3000~5000 RMB

○5001~8000 RMB

○8001~12000 RMB

○12001~18000 RMB

○18,000 RMB or more

10. Your type of residence is [single choice]*

◯Rural

◯Town

11. Has your daughter received self-funded vaccinations [multiple choice] *

○ Yes

○ No

12. Do any of your relatives have cancer [Single choice] *

○ Yes

○ No

13. Have you ever had an adverse reaction to a vaccination? [Multiple choice question] *

◯Never happened

◯Adverse reactions, fever, redness, swelling, hardness, etc.

◯Serious abnormal reaction, anaphylaxis, etc.

14. What kind of abnormal reaction to vaccination did your daughter have [multiple choice]*?

◯Never happened

◯Adverse reactions, fever, redness, swelling, hardness, etc.

◯Serious abnormal reaction, anaphylaxis, etc.

15. Does your daughter have a history of any of the following diseases: [multiple choice]*

Hypertension

Cardiovascular Disease

Epilepsy

Asthma

Acute and chronic urinary tract diseases

Drug or food allergies

Liver disease

History of long-term hormone use

Other ________________*

None

16. Do you have a family history of the disease [Single choice]*

○ No

○ Yes (please specify) ________________ *

17. Have you educated your daughter about sexuality [multiple choice]*

○ Yes

○ No

II. Vaccine program selection

**Question 1**	**HPV vaccine A**	**HPV vaccine B**
Protective effect	50%	75%
Safety	Very Good	Average
Effect duration	Lifelong	15 years
Location	Imported	Imported
Vaccine type	4 valent	9 valent
Full payment	4000RMB	400RMB

[Single-select matrix question] *

	Vaccine A	Vaccine B

If vaccinated for yourself (wife), which HPV vaccine would you prefer?		
If you were to vaccinate your daughter, which HPV vaccine would you prefer?		

**Question 2**	**HPV vaccine A**	**HPV vaccine B**
Protective effect	95%	50%
Safety	Average	Very Good
Effect duration	Lifelong	15 years
Location	Imported	Imported
Vaccine type	9 valent	4 valent
Full payment	1000RMB	400RMB

[Single-select matrix question] *

	Vaccine A	Vaccine B
If vaccinated for yourself (wife), which HPV vaccine would you prefer?		
If you were to vaccinate your daughter, which HPV vaccine would you prefer?		

**Question 3**	**HPV vaccine A**	**HPV vaccine B**
Protective effect	75%	95%
Safety	Good	Good
Effect duration	Lifelong	15 years
Location	Domestic	Imported
Vaccine type	9 valent	2 valent
Full payment	4000RMB	400RMB

[Single-select matrix question] *

	Vaccine A	Vaccine B
If vaccinated for yourself (wife), which HPV vaccine would you prefer?		
If you were to vaccinate your daughter, which HPV vaccine would you prefer?		

**Question 4**	**HPV vaccine A**	**HPV vaccine B**
Protective effect	50%	75%
Safety	Very good	Average
Effect duration	5 years	Lifelong
Location	Domestic	Imported
Vaccine type	9 valent	4 valent
Full payment	1000RMB	400RMB

[Single-select matrix question] *

	Vaccine A	Vaccine B
If vaccinated for yourself (wife), which HPV vaccine would you prefer?		
If you were to vaccinate your daughter, which HPV vaccine would you prefer?		

**Question 5**	**HPV vaccine A**	**HPV vaccine B**
Protective effect	95%	50%
Safety	Average	Good
Effect duration	5 years	15 years
Location	Domestic	Imported
Vaccine type	9 valent	2 valent
Full payment	400RMB	1000RMB

[Single-select matrix question] *

	Vaccine A	Vaccine B
If vaccinated for yourself (wife), which HPV vaccine would you prefer?		
If you were to vaccinate your daughter, which HPV vaccine would you prefer?		

**Question 6**	**HPV vaccine A**	**HPV vaccine B**
Protective effect	95%	75%
Safety	Good	Very good
Effect duration	15 years	5 years
Location	Imported	Imported
Vaccine type	4 valent	2 valent
Full payment	4000RMB	400RMB

[Single-select matrix question] *

	Vaccine A	Vaccine B
If vaccinated for yourself (wife), which HPV vaccine would you prefer?		
If you were to vaccinate your daughter, which HPV vaccine would you prefer?		

**Question 7**	**HPV vaccine A**	**HPV vaccine B**
Protective effect	50%	95%
Safety	Average	Very good
Effect duration	Lifelong	5 years
Location	Domestic	Domestic
Vaccine type	4 valent	2 valent
Full payment	2000RMB	4000RMB

[Single-select matrix question] *

	Vaccine A	Vaccine B
If vaccinated for yourself (wife), which HPV vaccine would you prefer?		
If you were to vaccinate your daughter, which HPV vaccine would you prefer?		

**Question 8**	**HPV vaccine A**	**HPV vaccine B**
Protective effect	95%	75%
Safety	Good	Very good
Effect duration	5 years	Lifelong
Location	Domestic	Imported
Vaccine type	4 valent	9 valent
Full payment	1000RMB	4000RMB

[Single-select matrix question] *

	Vaccine A	Vaccine B
If vaccinated for yourself (wife), which HPV vaccine would you prefer?		
If you were to vaccinate your daughter, which HPV vaccine would you prefer?		

**Question 9**	**HPV vaccine A**	**HPV vaccine B**
Protective effect	75%	50%
Safety	Very good	Good
Effect duration	15 years	5 years
Location	Domestic	Imported
Vaccine type	9 valent	2 valent
Full payment	1000RMB	4000RMB

[Single-select matrix question] *

	Vaccine A	Vaccine B
If vaccinated for yourself (wife), which HPV vaccine would you prefer?		
If you were to vaccinate your daughter, which HPV vaccine would you prefer?		

**Question 10**	**HPV vaccine A**	**HPV vaccine B**
Protective effect	95%	50%
Safety	Good	Average
Effect duration	Lifelong	5 years
Location	Domestic	Imported
Vaccine type	2 valent	9 valent
Full payment	400RMB	1000RMB

[Single-select matrix question] *

	Vaccine A	Vaccine B
If vaccinated for yourself (wife), which HPV vaccine would you prefer?		
If you were to vaccinate your daughter, which HPV vaccine would you prefer?		

**Question 11**	**HPV vaccine A**	**HPV vaccine B**
Protective effect	95%	50%
Safety	Good	Average
Effect duration	5 years	Lifelong
Location	Imported	Domestic
Vaccine type	4 valent	9 valent
Full payment	2000RMB	400RMB

[Single-select matrix question] *

	Vaccine A	Vaccine B
If vaccinated for yourself (wife), which HPV vaccine would you prefer?		
If you were to vaccinate your daughter, which HPV vaccine would you prefer?		

**Question 12**	**HPV vaccine A**	**HPV vaccine B**
Protective effect	95%	75%
Safety	Good	Very good
Effect duration	5 years	15 years
Location	Imported	Domestic
Vaccine type	9 valent	2 valent
Full payment	400RMB	2000RMB

[Single-select matrix question] *

	Vaccine A	Vaccine B
If vaccinated for yourself (wife), which HPV vaccine would you prefer?		
If you were to vaccinate your daughter, which HPV vaccine would you prefer?		

Name of investigator/questionnaire distributor [fill in the blank]

____

This concludes the questionnaire, thank you for your cooperation!
